# Whole-genome sequence of the non-rhizobial root nodule endophyte *Bosea* sp. strain 685 isolated from the root nodule of *Astragalus umbellatus* Bunge., growing on the Kamchatka Peninsula

**DOI:** 10.1128/mra.01033-23

**Published:** 2024-01-11

**Authors:** Polina Guro, Irina Kuznetsova, Anna Sazanova, Andrey Belimov, Vera Safronova

**Affiliations:** 1 All-Russia Research Institute for Agricultural Microbiology (ARRIAM), Saint Petersburg, Russia; The University of Arizona, Tucson, Arizona, USA

**Keywords:** *Bosea*, plant-microbe interaction

## Abstract

This study reports the whole-genome sequence of an endosymbiotic bacteria *Bosea* sp. strain 685, which was isolated from the root nodule of *Astragalus umbellatus* Bunge. in the Kamchatka Peninsula, Russia. The genome consists of one chromosome and one plasmid with a total length of 6,795,213 bp and 65.37% of GC content.

## ANNOUNCEMENT

**Fig 1:**
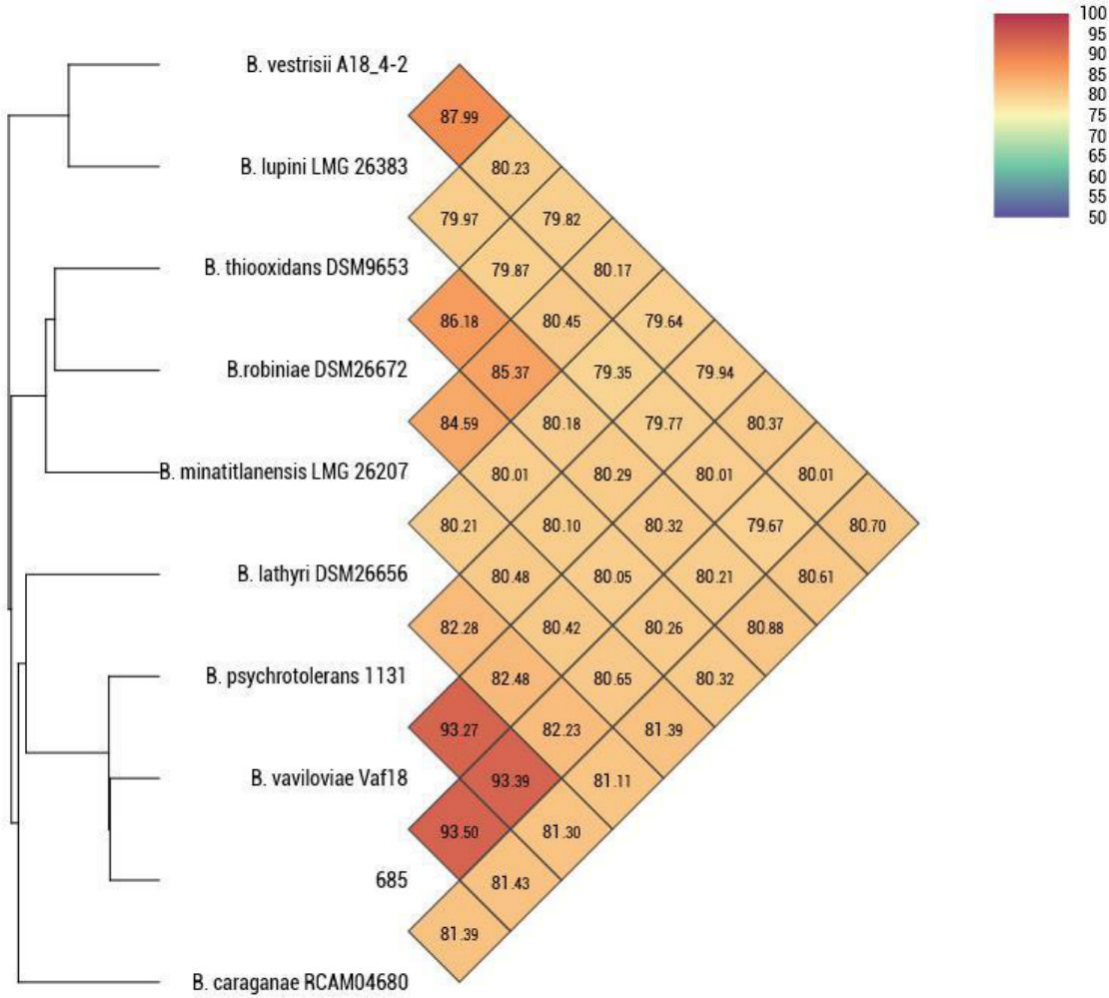
Heatmap of the orthologous average nucleotide identity between strain 685 and relative type strains from genus *Bosea*, calculated using OAT software.

The Kamchatka Peninsula lies at the intersection of two opposing climatic influences—continental subarctic and marine subarctic—and is characterized by a great diversity of tectonic landscapes (1). On the peninsula, plant growth is restricted by geography and a whole complex of limiting factors, making Kamchatka an interesting place to study plant-microbe interactions. Root nodules of *Astragalus umbellatus* were collected from the Kamchatka Peninsula (Russian Federation, 55.57201˚N, 159.45310˚E, h = 1,420 m). Individual nodules were sterilized for 1 minute in 70% ethanol, rinsed, homogenized in sterile tap water, and plated on YMSA agar plates (2). Colonies of strain 685 appeared after 6 days of incubation at 28°C. Single colonies were transferred to a new plate to obtain a pure culture. Isolates were stored at −80°C in an automated tube storage system (Liconic Instruments, Lichtenstein) at the Russian Collection of Agricultural Microorganisms. Genomic DNA without size selection was isolated using the DNeasy Blood & Tissue KIT (Qiagen, Germany) according to the manufacturer’s recommendations from a pure culture grown in liquid R2A broth at 28°C for 48 hours. The library was prepared using the SQK-LSK109 ligation sequencing kit and the EXP-NBD104 native barcoding expansion 1–12 kit (ONT, UK), skipping the DNA shearing step, and sequencing using a MinION sequencer with Flow Cell R9.4.1 (ONT). Basecalling was performed using Guppy (v.5.0.1, ONT). Sequencing resulted in 200,797 reads with an average read length of 8,565 bp. Quality control (QC) of the raw reads was performed using NanoStat (v.1.6.0) (3). The ONT reads were then filtered using the Filtlong tool (v.0.2.1, https://github.com/rrwick/Filtlong), with options --min_length 1000 and --keep_percent 95. QC after filtering resulted in 124,648 reads and an average read length of 13,097 bp. *De novo* assembly was performed using Flye (v.2.9) (4). Two circular contigs—a chromosome of 6,582,294-bp length and one plasmid of 212,919-bp length—were assembled with a total size of 6.7 Mbp and an average coverage of 242× with no specific start point for the assembled contigs. To generate consensus sequences, we polished the resulting assembly with a Medaka (v.1.8.0, https://github.com/nanoporetech/medaka) using raw reads. Default parameters were used for all software unless otherwise noted. Assembly statistics were provided by QUAST (v.5.0.2) (5). The GC content was 65.37%, and the N_50_ value of the assembled genome was 6,582,294 bp. The completeness of the assemblies was measured using BUSCO (v.5.4.4) with the rhizobiales_odb10 data set and gave the following estimates of completeness of 99.2% (6). The taxonomic rank was determined by 16s rRNA (*rrs*) BLASTn comparison, and average nucleotide identity (ANI) analysis was calculated using OrthoANI by OAT software (v.0.93.1) (7, 8). Strain 685 was most closely related to the type strain *Bosea vaviloviae* Vaf18 (GCF_001741865.1; CP017147.1) with 93.50% average nucleotide identity (Fig. 1) and 99.80% *rrs* identity, on which basis strain 685 was assigned to *Bosea* sp. Annotation of the assembly using PGAP (v.6.6) resulted in 6,262 protein-coding genes, 86 pseudogenes, which can be explained by using only one sequencing kit, and 59 RNA genes (6 rRNAs, 49 tRNAs, and 4 ncRNAs) (9).

## Data Availability

All data are available at GenBank under BioProject accession number PRJNA1018331. The BioSample accession number is SAMN37432815. The raw MinION data can be found under number SRR26086523 (raw reads deposited at Sequence Read Archive without quality processing) and the assembly accession number CP134778.1-CP134779.1
